# 2,2′-Dimethyl-1,1′-[2,2-bis­(bromo­methyl)propane-1,3-di­yl]dibenzimidazole hemihydrate

**DOI:** 10.1107/S1600536812007751

**Published:** 2012-02-24

**Authors:** Xing Wang, Chun-Bo Liu, Shen-Tang Wang, Yong-Sheng Yan, Ling Liu

**Affiliations:** a School of Chemistry and Chemical Engineering, Jiangsu University, Zhenjiang 212013, People’s Republic of China

## Abstract

The title compound, C_21_H_22_Br_2_N_4_·0.5H_2_O, contains two benzimidazole groups which may provide two potential coordination nodes for the construction of metal–organic frameworks. The mean planes of the two imidazole groups are almost perpendicular, with a dihedral angle of 83.05 (2)°, and adjacent mol­ecules are linked into a one-dimensional chain by π–π stacking inter­actions between imidazole groups of different mol­ecules [centroid-to-centroid distances of 3.834 (2) and 3.522 (2) Å].

## Related literature

For preparation of the *N*-donor compound, see: Bai *et al.* (2010 ▶). For a related structure, see: Wei *et al.* (2011 ▶). For constructions and applications of metal–organic frameworks, see: Kuppler *et al.* (2009 ▶); Wang *et al.* (2011 ▶).
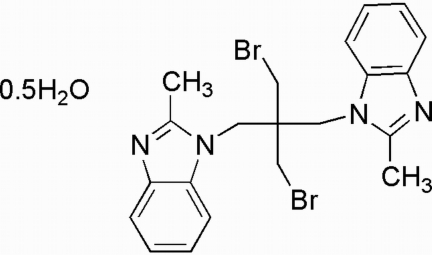


## Experimental

### 

#### Crystal data

C_21_H_22_Br_2_N_4_·0.5H_2_O
*M* *_r_* = 499.26
Monoclinic, 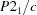

*a* = 12.647 (3) Å
*b* = 8.1065 (16) Å
*c* = 20.580 (4) Å
β = 107.42 (3)°
*V* = 2013.1 (7) Å^3^
*Z* = 4
Mo *K*α radiation
μ = 4.04 mm^−1^
*T* = 153 K
0.15 × 0.11 × 0.10 mm

#### Data collection

Rigaku Saturn 724+ CCD diffractometer
Absorption correction: multi-scan (*CrystalClear*; Rigaku, 2007 ▶) *T*_min_ = 0.582, *T*_max_ = 0.688
9078 measured reflections
3662 independent reflections
2786 reflections with *I* > 2σ(*I*)
*R*_int_ = 0.059

#### Refinement

*R*[*F* ^2^ > 2σ(*F* ^2^)] = 0.074
*wR*(*F* ^2^) = 0.159
*S* = 1.13
3662 reflections
253 parameters
H-atom parameters constrained
Δρ_max_ = 1.00 e Å^−3^
Δρ_min_ = −0.71 e Å^−3^

### 

Data collection: *CrystalClear* (Rigaku, 2007 ▶); cell refinement: *CrystalClear*; data reduction: *CrystalClear*; program(s) used to solve structure: *SHELXS97* (Sheldrick, 2008 ▶); program(s) used to refine structure: *SHELXL97* (Sheldrick, 2008 ▶); molecular graphics: *CrystalClear* and *DIAMOND* (Brandenburg, 1999 ▶); software used to prepare material for publication: *SHELXTL* (Sheldrick, 2008 ▶).

## Supplementary Material

Crystal structure: contains datablock(s) global, I. DOI: 10.1107/S1600536812007751/bg2435sup1.cif

Structure factors: contains datablock(s) I. DOI: 10.1107/S1600536812007751/bg2435Isup2.hkl

Supplementary material file. DOI: 10.1107/S1600536812007751/bg2435Isup3.cml

Additional supplementary materials:  crystallographic information; 3D view; checkCIF report

## Figures and Tables

**Fig. 1.**
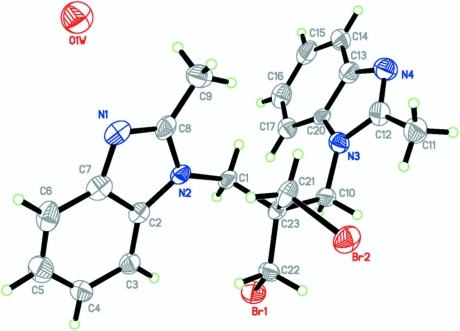
ORTEP plot of the title compound (50% probability ellipsoids). H atoms on the water molecule are not included in the model.

**Fig. 2.**
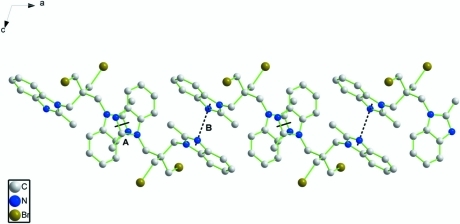
A view of the 1D chain structure formed by the π–π stacking interactions through b-axis. All the H atoms and water molecules were omitted for clarity. Intercentroid distances are A: 3.834 (2); B: 3.522 (2) Å.

## supplementary crystallographic information

### Comment

Metal–organic frameworks have gained more and more attention not only because of their intriguing structures, but also because of their potential applications as functional materials (Kuppler *et al.* 2009). In general, noncovalent interactions such as π–π stacking, can be used to direct the supramolecular architectures (Wang *et al.* 2011). So the N-donor title compound (L) is expected to be a good choice for the construction of metal–organic frameworks, mainly because the two benzimidazol N atoms can be potentially active coordination sites (Bai *et al.* 2010), while the two planar groups in turn can freely twist around the quaternary C atom and the two -CH_2_- groups so as to match the requirements of various coordination geometries (Wei *et al.* 2011). In addition, π-=π interaction may occur between benzimidazol groups from different L molecules, to promote a supramolecular assembly.

Fig. 1 shows an ellipsoid plot of the asymmetric unit of (L). The dihedral angle between the mean planes of the two imidazol rings is 83.05 (2)°. In addition, π–π stacking interactions between imidazole groups from adjacent molecules (intercentroid distances: A: 3.834 (2) and B: 3.522 (2) Å) connect them into a 1D chain structure (Fig. 2).

### Experimental

The synthesis, initially aimed to produce a Cd complex, followed a previous literature procedure (Bai *et al.* 2010). However, the X-ray crystallographic study confirmed that the cation did not enter into the structure and the product corresponded to the title compound. A mixture of CdCl_2_ (0.0183 g, 0.1 mmol), L (0.0491 g, 0.1 mmol) and water (15 ml) was stirred for one hour, and then transferred to a 25 ml Teflon-lined stainless steel reactor. The reactor was heated to 433 K for 72 h, and cooled to room temperature in the autogenous conditions. Colourless block crystals of (L) were obtained with a yield of 65%.

### Refinement

H atoms attached to carbon were placed in calculated positions and refined as riding, with *U*_iso_(H) = x *U*_eq_(C) (C—H (methyl): 0.96 Å, x = 1.5; C—H (aromatic): 0.93 Å; x = 1.2; C—H (methylene): 0.97 Å, x = 1.2). The O atom of the water solvate is disordered around an inversion centre, for what its site occupation factor is 0.5. Its H atoms could not be found in the difference map.

### Crystal data

**Table:**

C_21_H_22_Br_2_N_4_·0.5H_2_O	*F*(000) = 1004
*M**_r_* = 499.26	*D*_x_ = 1.647 Mg m^−^^3^
Monoclinic, *P*2_1_/*c*	Mo *K*α radiation, λ = 0.71073 Å
Hall symbol: -P 2ybc	Cell parameters from 6583 reflections
*a* = 12.647 (3) Å	θ = 2.5–29.1°
*b* = 8.1065 (16) Å	µ = 4.04 mm^−^^1^
*c* = 20.580 (4) Å	*T* = 153 K
β = 107.42 (3)°	Prism, colourless
*V* = 2013.1 (7) Å^3^	0.15 × 0.11 × 0.10 mm
*Z* = 4	

### Data collection

**Table:**

Rigaku Saturn 724+ CCD diffractometer	3662 independent reflections
Radiation source: fine-focus sealed tube	2786 reflections with *I* > 2σ(*I*)
Graphite monochromator	*R*_int_ = 0.059
Detector resolution: 28.5714 pixels mm^-1^	θ_max_ = 25.4°, θ_min_ = 3.0°
ω scans	*h* = −14→15
Absorption correction: multi-scan (*CrystalClear*; Rigaku, 2007)	*k* = −9→9
*T*_min_ = 0.582, *T*_max_ = 0.688	*l* = −20→24
9078 measured reflections	

### Refinement

**Table:**

Refinement on *F*^2^	Primary atom site location: structure-invariant direct methods
Least-squares matrix: full	Secondary atom site location: difference Fourier map
*R*[*F*^2^ > 2σ(*F*^2^)] = 0.074	Hydrogen site location: inferred from neighbouring sites
*wR*(*F*^2^) = 0.159	H-atom parameters constrained
*S* = 1.13	*w* = 1/[σ^2^(*F*_o_^2^) + (0.053*P*)^2^ + 5.0634*P*] where *P* = (*F*_o_^2^ + 2*F*_c_^2^)/3
3662 reflections	(Δ/σ)_max_ = 0.001
253 parameters	Δρ_max_ = 1.00 e Å^−^^3^
0 restraints	Δρ_min_ = −0.71 e Å^−^^3^

### Special details

**Table:**

Geometry. All esds (except the esd in the dihedral angle between two l.s. planes) are estimated using the full covariance matrix. The cell esds are taken into account individually in the estimation of esds in distances, angles and torsion angles; correlations between esds in cell parameters are only used when they are defined by crystal symmetry. An approximate (isotropic) treatment of cell esds is used for estimating esds involving l.s. planes.
Refinement. Refinement of F^2^ against ALL reflections. The weighted R-factor wR and goodness of fit S are based on F^2^, conventional R-factors R are based on F, with F set to zero for negative F^2^. The threshold expression of F^2^ > 2sigma(F^2^) is used only for calculating R-factors(gt) etc. and is not relevant to the choice of reflections for refinement. R-factors based on F^2^ are statistically about twice as large as those based on F, and R-factors based on ALL data will be even larger.

### Fractional atomic coordinates and isotropic or equivalent isotropic displacement parameters (Å^2^)

**Table:**

	*x*	*y*	*z*	*U*_iso_*/*U*_eq_	Occ. (<1)
C1	0.6424 (6)	0.2016 (8)	0.9301 (3)	0.0256 (16)	
H1A	0.7009	0.1863	0.9726	0.031*	
H1B	0.6036	0.3025	0.9342	0.031*	
C2	0.4530 (6)	0.0612 (8)	0.8838 (3)	0.0242 (15)	
C3	0.3807 (6)	0.1770 (9)	0.8446 (3)	0.0297 (17)	
H3	0.4043	0.2829	0.8386	0.036*	
C4	0.2721 (6)	0.1286 (10)	0.8149 (4)	0.0364 (18)	
H4	0.2216	0.2040	0.7888	0.044*	
C5	0.2365 (7)	−0.0308 (10)	0.8233 (4)	0.042 (2)	
H5	0.1634	−0.0600	0.8014	0.051*	
C6	0.3062 (7)	−0.1452 (10)	0.8627 (4)	0.039 (2)	
H6	0.2813	−0.2502	0.8690	0.047*	
C7	0.4159 (6)	−0.0989 (9)	0.8933 (3)	0.0308 (17)	
C8	0.5876 (6)	−0.0897 (9)	0.9525 (4)	0.0308 (17)	
C9	0.6978 (6)	−0.1342 (10)	0.9993 (4)	0.0386 (19)	
H9A	0.7479	−0.0432	1.0028	0.058*	
H9B	0.7259	−0.2289	0.9818	0.058*	
H9C	0.6911	−0.1592	1.0434	0.058*	
C10	0.8018 (5)	0.3379 (8)	0.8972 (3)	0.0286 (16)	
H10A	0.8364	0.3435	0.8611	0.034*	
H10B	0.7769	0.4483	0.9034	0.034*	
C11	1.0146 (6)	0.1356 (10)	0.9069 (4)	0.0401 (19)	
H11A	0.9545	0.1503	0.8658	0.060*	
H11B	1.0283	0.0200	0.9153	0.060*	
H11C	1.0800	0.1877	0.9020	0.060*	
C12	0.9851 (6)	0.2113 (9)	0.9649 (4)	0.0354 (18)	
C13	0.9953 (6)	0.3007 (9)	1.0651 (4)	0.0327 (17)	
C14	1.0284 (6)	0.3408 (10)	1.1335 (4)	0.0362 (18)	
H14	1.0959	0.3042	1.1623	0.043*	
C15	0.9585 (7)	0.4364 (10)	1.1578 (4)	0.044 (2)	
H15	0.9791	0.4651	1.2037	0.052*	
C16	0.8556 (7)	0.4915 (9)	1.1138 (4)	0.0390 (19)	
H16	0.8099	0.5572	1.1310	0.047*	
C17	0.8226 (6)	0.4492 (8)	1.0462 (4)	0.0300 (17)	
H17	0.7551	0.4853	1.0172	0.036*	
C20	0.8938 (6)	0.3495 (8)	1.0220 (3)	0.0258 (16)	
C21	0.7252 (6)	0.0544 (9)	0.8506 (4)	0.0324 (18)	
H21A	0.6603	−0.0156	0.8428	0.039*	
H21B	0.7833	0.0069	0.8880	0.039*	
C22	0.6175 (6)	0.3104 (9)	0.8090 (3)	0.0302 (17)	
H22A	0.5539	0.2397	0.7899	0.036*	
H22B	0.6553	0.3245	0.7747	0.036*	
C23	0.6969 (5)	0.2250 (8)	0.8721 (3)	0.0225 (15)	
N1	0.5029 (5)	−0.1870 (7)	0.9376 (3)	0.0305 (14)	
N2	0.5642 (5)	0.0633 (7)	0.9216 (3)	0.0250 (13)	
N3	0.8870 (5)	0.2914 (7)	0.9599 (3)	0.0296 (14)	
N4	1.0516 (5)	0.2108 (8)	1.0281 (3)	0.0378 (16)	
O1W	0.5525 (10)	−0.4843 (13)	1.0222 (6)	0.055 (3)	0.50
Br1	0.56570 (7)	0.52696 (9)	0.83015 (4)	0.0391 (3)	
Br2	0.77367 (7)	0.05147 (10)	0.76915 (4)	0.0389 (3)	

### Atomic displacement parameters (Å^2^)

**Table:**

	*U*^11^	*U*^22^	*U*^33^	*U*^12^	*U*^13^	*U*^23^
C1	0.029 (4)	0.022 (3)	0.026 (4)	−0.006 (3)	0.009 (3)	0.005 (3)
C2	0.032 (4)	0.021 (4)	0.021 (4)	−0.003 (3)	0.011 (3)	−0.005 (3)
C3	0.031 (4)	0.037 (4)	0.023 (4)	−0.002 (3)	0.010 (3)	0.000 (3)
C4	0.029 (4)	0.050 (5)	0.028 (4)	−0.001 (4)	0.005 (3)	0.003 (4)
C5	0.041 (5)	0.051 (5)	0.031 (4)	−0.015 (4)	0.005 (4)	−0.010 (4)
C6	0.045 (5)	0.039 (5)	0.036 (5)	−0.020 (4)	0.016 (4)	−0.014 (4)
C7	0.044 (5)	0.024 (4)	0.026 (4)	−0.003 (3)	0.013 (4)	−0.008 (3)
C8	0.036 (4)	0.032 (4)	0.033 (4)	0.004 (3)	0.023 (4)	0.004 (3)
C9	0.038 (5)	0.041 (5)	0.035 (4)	0.008 (4)	0.009 (4)	0.003 (4)
C10	0.026 (4)	0.029 (4)	0.028 (4)	−0.006 (3)	0.004 (3)	0.006 (3)
C11	0.038 (5)	0.049 (5)	0.036 (5)	0.002 (4)	0.015 (4)	−0.001 (4)
C12	0.033 (4)	0.039 (4)	0.037 (5)	−0.003 (4)	0.014 (4)	−0.001 (4)
C13	0.032 (4)	0.036 (4)	0.029 (4)	−0.007 (3)	0.008 (4)	−0.002 (3)
C14	0.029 (4)	0.044 (5)	0.034 (4)	−0.004 (4)	0.007 (4)	0.001 (4)
C15	0.046 (5)	0.052 (5)	0.034 (5)	−0.020 (4)	0.013 (4)	0.002 (4)
C16	0.044 (5)	0.035 (4)	0.041 (5)	−0.013 (4)	0.017 (4)	−0.008 (4)
C17	0.031 (4)	0.020 (4)	0.039 (4)	−0.005 (3)	0.011 (4)	0.000 (3)
C20	0.024 (4)	0.032 (4)	0.018 (4)	−0.012 (3)	0.002 (3)	0.005 (3)
C21	0.043 (5)	0.031 (4)	0.031 (4)	−0.006 (3)	0.024 (4)	−0.004 (3)
C22	0.024 (4)	0.043 (4)	0.021 (4)	0.004 (3)	0.004 (3)	0.000 (3)
C23	0.026 (4)	0.020 (3)	0.023 (4)	−0.003 (3)	0.009 (3)	0.000 (3)
N1	0.047 (4)	0.021 (3)	0.028 (3)	0.000 (3)	0.017 (3)	−0.003 (3)
N2	0.030 (3)	0.028 (3)	0.019 (3)	−0.002 (3)	0.010 (3)	0.001 (2)
N3	0.025 (3)	0.027 (3)	0.035 (4)	−0.003 (3)	0.006 (3)	0.002 (3)
N4	0.037 (4)	0.041 (4)	0.035 (4)	0.006 (3)	0.010 (3)	0.002 (3)
O1W	0.062 (8)	0.031 (6)	0.066 (9)	−0.007 (6)	0.012 (7)	0.014 (6)
Br1	0.0404 (5)	0.0328 (5)	0.0436 (5)	0.0076 (3)	0.0121 (4)	0.0086 (3)
Br2	0.0469 (5)	0.0441 (5)	0.0302 (4)	0.0022 (4)	0.0184 (4)	−0.0040 (3)

### Geometric parameters (Å, º)

**Table:**

C1—N2	1.470 (8)	C11—H11A	0.9600
C1—C23	1.559 (9)	C11—H11B	0.9600
C1—H1A	0.9700	C11—H11C	0.9600
C1—H1B	0.9700	C12—N4	1.321 (9)
C2—C3	1.388 (9)	C12—N3	1.377 (9)
C2—N2	1.389 (9)	C13—C20	1.381 (10)
C2—C7	1.413 (9)	C13—C14	1.383 (10)
C3—C4	1.382 (10)	C13—N4	1.393 (9)
C3—H3	0.9300	C14—C15	1.377 (11)
C4—C5	1.396 (11)	C14—H14	0.9300
C4—H4	0.9300	C15—C16	1.417 (11)
C5—C6	1.367 (11)	C15—H15	0.9300
C5—H5	0.9300	C16—C17	1.372 (10)
C6—C7	1.392 (10)	C16—H16	0.9300
C6—H6	0.9300	C17—C20	1.408 (10)
C7—N1	1.397 (9)	C17—H17	0.9300
C8—N1	1.291 (9)	C20—N3	1.341 (8)
C8—N2	1.386 (9)	C21—C23	1.526 (9)
C8—C9	1.481 (10)	C21—Br2	1.951 (7)
C9—H9A	0.9600	C21—H21A	0.9700
C9—H9B	0.9600	C21—H21B	0.9700
C9—H9C	0.9600	C22—C23	1.547 (9)
C10—N3	1.460 (8)	C22—Br1	1.968 (7)
C10—C23	1.567 (9)	C22—H22A	0.9700
C10—H10A	0.9700	C22—H22B	0.9700
C10—H10B	0.9700	O1W—O1W^i^	1.39 (2)
C11—C12	1.486 (10)		
			
N2—C1—C23	115.9 (5)	N4—C12—C11	123.5 (7)
N2—C1—H1A	108.3	N3—C12—C11	124.9 (7)
C23—C1—H1A	108.3	C20—C13—C14	121.8 (7)
N2—C1—H1B	108.3	C20—C13—N4	109.2 (6)
C23—C1—H1B	108.3	C14—C13—N4	129.1 (7)
H1A—C1—H1B	107.4	C15—C14—C13	118.1 (7)
C3—C2—N2	134.2 (6)	C15—C14—H14	121.0
C3—C2—C7	120.9 (7)	C13—C14—H14	121.0
N2—C2—C7	104.9 (6)	C14—C15—C16	120.8 (8)
C4—C3—C2	117.3 (7)	C14—C15—H15	119.6
C4—C3—H3	121.3	C16—C15—H15	119.6
C2—C3—H3	121.3	C17—C16—C15	120.8 (8)
C3—C4—C5	121.5 (7)	C17—C16—H16	119.6
C3—C4—H4	119.2	C15—C16—H16	119.6
C5—C4—H4	119.2	C16—C17—C20	118.0 (7)
C6—C5—C4	121.8 (7)	C16—C17—H17	121.0
C6—C5—H5	119.1	C20—C17—H17	121.0
C4—C5—H5	119.1	N3—C20—C13	107.1 (6)
C5—C6—C7	117.6 (7)	N3—C20—C17	132.4 (7)
C5—C6—H6	121.2	C13—C20—C17	120.5 (6)
C7—C6—H6	121.2	C23—C21—Br2	114.9 (5)
C6—C7—N1	129.8 (7)	C23—C21—H21A	108.5
C6—C7—C2	120.8 (7)	Br2—C21—H21A	108.5
N1—C7—C2	109.3 (6)	C23—C21—H21B	108.5
N1—C8—N2	112.9 (6)	Br2—C21—H21B	108.5
N1—C8—C9	123.9 (7)	H21A—C21—H21B	107.5
N2—C8—C9	123.1 (7)	C23—C22—Br1	112.9 (4)
C8—C9—H9A	109.5	C23—C22—H22A	109.0
C8—C9—H9B	109.5	Br1—C22—H22A	109.0
H9A—C9—H9B	109.5	C23—C22—H22B	109.0
C8—C9—H9C	109.5	Br1—C22—H22B	109.0
H9A—C9—H9C	109.5	H22A—C22—H22B	107.8
H9B—C9—H9C	109.5	C21—C23—C22	108.2 (6)
N3—C10—C23	118.0 (5)	C21—C23—C1	107.9 (5)
N3—C10—H10A	107.8	C22—C23—C1	111.9 (5)
C23—C10—H10A	107.8	C21—C23—C10	112.1 (6)
N3—C10—H10B	107.8	C22—C23—C10	106.8 (5)
C23—C10—H10B	107.8	C1—C23—C10	110.0 (5)
H10A—C10—H10B	107.1	C8—N1—C7	106.2 (6)
C12—C11—H11A	109.5	C8—N2—C2	106.7 (5)
C12—C11—H11B	109.5	C8—N2—C1	125.7 (6)
H11A—C11—H11B	109.5	C2—N2—C1	127.5 (6)
C12—C11—H11C	109.5	C20—N3—C12	107.1 (6)
H11A—C11—H11C	109.5	C20—N3—C10	124.8 (6)
H11B—C11—H11C	109.5	C12—N3—C10	126.8 (6)
N4—C12—N3	111.7 (6)	C12—N4—C13	104.9 (6)
			
N2—C2—C3—C4	−178.8 (7)	N3—C10—C23—C21	−66.2 (8)
C7—C2—C3—C4	−0.7 (10)	N3—C10—C23—C22	175.5 (6)
C2—C3—C4—C5	−0.7 (10)	N3—C10—C23—C1	53.8 (8)
C3—C4—C5—C6	2.0 (12)	N2—C8—N1—C7	0.7 (8)
C4—C5—C6—C7	−1.7 (12)	C9—C8—N1—C7	179.1 (6)
C5—C6—C7—N1	177.4 (7)	C6—C7—N1—C8	−179.0 (7)
C5—C6—C7—C2	0.3 (11)	C2—C7—N1—C8	−1.6 (8)
C3—C2—C7—C6	1.0 (10)	N1—C8—N2—C2	0.5 (8)
N2—C2—C7—C6	179.5 (6)	C9—C8—N2—C2	−178.0 (6)
C3—C2—C7—N1	−176.7 (6)	N1—C8—N2—C1	178.2 (6)
N2—C2—C7—N1	1.9 (7)	C9—C8—N2—C1	−0.2 (10)
C20—C13—C14—C15	−2.1 (11)	C3—C2—N2—C8	176.8 (7)
N4—C13—C14—C15	178.0 (7)	C7—C2—N2—C8	−1.4 (7)
C13—C14—C15—C16	0.2 (11)	C3—C2—N2—C1	−0.8 (12)
C14—C15—C16—C17	0.8 (11)	C7—C2—N2—C1	−179.1 (6)
C15—C16—C17—C20	0.1 (10)	C23—C1—N2—C8	99.7 (7)
C14—C13—C20—N3	−179.9 (7)	C23—C1—N2—C2	−83.0 (8)
N4—C13—C20—N3	0.1 (8)	C13—C20—N3—C12	−1.5 (7)
C14—C13—C20—C17	3.0 (10)	C17—C20—N3—C12	175.1 (7)
N4—C13—C20—C17	−177.0 (6)	C13—C20—N3—C10	−169.2 (6)
C16—C17—C20—N3	−178.2 (7)	C17—C20—N3—C10	7.4 (11)
C16—C17—C20—C13	−1.9 (10)	N4—C12—N3—C20	2.6 (8)
Br2—C21—C23—C22	49.9 (7)	C11—C12—N3—C20	−176.8 (7)
Br2—C21—C23—C1	171.1 (5)	N4—C12—N3—C10	169.9 (6)
Br2—C21—C23—C10	−67.6 (7)	C11—C12—N3—C10	−9.5 (11)
Br1—C22—C23—C21	176.2 (4)	C23—C10—N3—C20	−94.6 (8)
Br1—C22—C23—C1	57.5 (6)	C23—C10—N3—C12	100.1 (8)
Br1—C22—C23—C10	−62.9 (6)	N3—C12—N4—C13	−2.4 (8)
N2—C1—C23—C21	−40.4 (8)	C11—C12—N4—C13	177.0 (7)
N2—C1—C23—C22	78.5 (7)	C20—C13—N4—C12	1.5 (8)
N2—C1—C23—C10	−162.9 (5)	C14—C13—N4—C12	−178.6 (8)

Symmetry code: (i) −*x*+1, −*y*−1, −*z*+2.
